# Molecular and Clinical Profiles of Pediatric Monogenic Diabetes Subtypes: Comprehensive Genetic Analysis of 138 Patients

**DOI:** 10.1210/clinem/dgae779

**Published:** 2024-11-06

**Authors:** Qiaoli Zhou, Sama Samadli, Haoyu Zhang, Xueqin Zheng, Bixia Zheng, Aihua Zhang, Wei Gu

**Affiliations:** Department of Endocrinology, Children’s Hospital of Nanjing Medical University, Nanjing, 210008, China; Nanjing Key Laboratory of Pediatrics, Children’s Hospital of Nanjing Medical University, Nanjing, 210008, China; Department of Pediatric Diseases II, Azerbaijan Medical University, Baku, AZ1022, Azerbaijan; Department of Endocrinology, Children’s Hospital of Nanjing Medical University, Nanjing, 210008, China; Department of Endocrinology, Children’s Hospital of Nanjing Medical University, Nanjing, 210008, China; Nanjing Key Laboratory of Pediatrics, Children’s Hospital of Nanjing Medical University, Nanjing, 210008, China; Nanjing Key Laboratory of Pediatrics, Children’s Hospital of Nanjing Medical University, Nanjing, 210008, China; Department of Endocrinology, Children’s Hospital of Nanjing Medical University, Nanjing, 210008, China

**Keywords:** monogenic diabetes, maturity onset diabetes of the young, MODY, neonatal diabetes, syndromic monogenic diabetes, next-generation sequencing

## Abstract

**Background:**

Single gene variants that give rise to neonatal diabetes mellitus (NDM), maturity onset diabetes of the young (MODY), and syndromic forms of diabetes mellitus (SDM) are responsible for 3.1% to 4.2% of all diabetes cases. This single-center study with a relatively larger sample size aimed to evaluate the clinical and genetic characteristics of Chinese children with suspected monogenic diabetes (MD) using next-generation sequencing (NGS) methods.

**Materials and Methods:**

Data were collected from 1550 consecutive children diagnosed with diabetes/hyperglycemia at the Endocrinology Department of Children's Hospital of Nanjing Medical University from 2012 to 2023. The genotype and phenotype of 138 children with suspected MD were retrospectively analyzed.

**Results:**

Among 138 children, 16, 97, and 25 patients with NDM, suspected MODY, and SDM, respectively, were assessed by NGS, with a pick-up rate of 87.5%, 57.8%, and 56%, respectively. In total, there was a high pick-up rate of MD, with 58% (80 of 138) among antibody-negative pediatric patients. Pathogenic variants were found in *GCK, HNF1A, INS, KCNJ11, INSR, HNF4A, ABCC8, WFS1, ALMS1, HNF1B, BLK,* and *ZFP57* genes with 13 novel variants in addition to 4 patients with copy number variants. In this cohort, *GCK-*MODY was the leading cause and the mildest type of MODY. *GCK*-MODY displayed favorable lipid profile when compared to non-*GCK*-MODY and MODYX, which might be cardioprotective. Following an accurate genetic diagnosis of diabetes, 19 patients switched from insulin therapy to oral agents or lifestyle interventions.

**Conclusion:**

NGS tests helped to identify the precise etiology of monogenic diabetic patients, which has implications for better individualized management.

Single gene mutations that give rise to neonatal diabetes mellitus (NDM), maturity onset diabetes of the young (MODY), and syndromic forms of diabetes (SDM) are responsible for 3.1% to 4.2% of all diabetes cases according to the results of the nationwide population studies (1, 2). However, among antibody-negative children with diabetes, the proportion of monogenic diabetes (MD) reaches as high as to 12.5% (3). Detailed analysis of the TODAY clinical trial revealed that at least 4.5% of adolescents diagnosed with type 2 diabetes mellitus (T2DM) are actually having MODY (4). A multicenter study in China estimated that this number might be 6% among the children diagnosed with type 1 diabetes mellitus (T1DM) (5). Increasing awareness and facilitated access to the genetic tests allow the identification of the more monogenic cases and the lesser misdiagnosis as T1DM or T2DM. Notably, in the UK, the Genetic Diabetes Nurse Education Initiative, coupled with the integration of advanced sequencing techniques and the MODY probability calculator into clinical practice, tripled the detection rate of MODY within a decade (6).

Next-generation sequencing (NGS) techniques are the game-changer in terms of accurate diagnosis of MD, which allows for the most appropriate treatment and more precise prognostics (7). Of note, an established diagnosis of *GCK*-MODY enables a switch from unnecessary insulin injections to lifestyle modifications (8). While diabetes arising from *ABCC8* and *KCNJ11* requires high-dose sulfonylureas (9), a low-dose sulfonylureas is sufficient to obtain good control of blood glucose levels in the majority of *HNF4A*-MODY and *HNF1A*-MODY patients (10). Besides, genetic diagnosis of MD not only provides a foundation for a gene-based approach but also a variant-based approach toward the clinical heterogeneity and treatment options for MD patients. For instance, the type and the location of mutation might be associated with the clinical manifestations, age at onset, and disease severity (11). Some variants to *ABCC8* and *KCNJ11* genes may render K^+^ channel less sensitive to sulfonylureas (9). Furthermore, the identified causative genes are the best predictor of possible complications and additional clinical phenotypes as well as future incidences among family members (12). Finally, NGS has led to the discovery of candidate genes that were not previously considered causal for MD. In this regard, 7 genes have been added to the MD list in the recent years (13), and many are expected to be discovered in the future.

Although nationwide and multinational prospective and retrospective studies in the pediatric patients with MODY are available in the literature, distinct eligibility criteria and testing of only a few MODY-related genes make it difficult to determine the true prevalence of MD and its subtypes in different populations (1, 2, 14). Earlier multinational studies urged to conclude that K^+^ channel mutations are the leading cause of neonatal diabetes (15). In contrast, subsequent single-center studies from non-Caucasian ethnicities demonstrated predominance of non-K^+^ channel mutations (16, 17). Additionally, the incidence, clinical, and molecular characteristics of SDM and its subtypes are largely unknown. Lastly, the high rate of unyielded genetic test results among patients with MD, especially when a limited number of genes are tested, makes the comprehensive genomic testing methods such as whole-exome sequencing (WES) even more indispensable. Taking into account these research gaps, our single-center study with a relatively larger sample size aimed to evaluate the genetic and clinical characteristics of Eastern Chinese children with suspected MD using NGS methods.

## Materials and Methods

### Subjects

We conducted a single-center retrospective study of 1550 consecutive children diagnosed with diabetes or hyperglycemia at the Endocrinology Department of Children's Hospital of Nanjing Medical University from 2012 to 2023. Onset of diabetes or hyperglycemia was under 18 years of age in all patients. Diabetes mellitus and hyperglycemia (impaired fasting glucose and/or impaired glucose tolerance) were defined in accordance with the criteria established by the American Diabetes Association. Impaired fasting glucose is defined as a fasting plasma glucose level from 5.6 to 6.9 mmol/L and impaired glucose tolerance as a 2-hour plasma glucose level during an oral glucose tolerance test (OGTT) from 7.8 to 11.0 mmol/L. The diagnostic criteria for diabetes encompass a fasting plasma glucose level of ≥7.0 mmol/L, a 2-hour plasma glucose level of ≥11.1 mmol/L, or a random plasma glucose level of ≥11.1 mmol/L combined with typical symptoms of hyperglycemia or hyperglycemic crisis. For all patients, the following clinical data were gathered: age at onset, sex, family history of diabetes mellitus, birthweight (BW), anthropometric measurements [height, weight, and body mass index (BMI)], laboratory blood tests [plasma glucose, C-peptide, insulin, glycated hemoglobin (HbA1c)], β-cell antibodies (ICA, GADA, IA-2A, IAA, and ZnT8A), lipid markers [triglyceride (TG), total cholesterol (TC), high-density lipoprotein (HDL)], and presence of acanthosis nigricans and extrapancreatic features (ie, deafness, retinopathy, renal abnormality, dysmorphic features, development delay, anemia etc.). The classification of diabetes for the 1550 patients was established based on their clinical characteristics following the American Diabetes Association guidelines. Cases of T1DM, T2DM, secondary diabetes due to drugs and endocrinopathies, and mitochondrial diabetes were excluded. A total of 165 patients were identified as having suspected monogenic diabetes, characterized by the absence of β-cell antibodies and meeting at least 1 of the following 6 specific criteria:

1. Diagnosis of diabetes or hyperglycemia before 12 months of age
2. Positive family history of nonautoimmune diabetes mellitus or hyperglycemia in 1 or more generations of first-degree relatives
3. Preserved beta-cell function with fasting C-peptide ≥0.2 nmol/L or stimulated C-peptide ≥0.6 nmol/L for over 3 years after the onset of diabetes
4. Stable, mild fasting hyperglycemia (5.6-8.5 mmol/L)
5. Insulin resistance without obesity or severe insulin resistance associated with disproportionate obesity (insulin resistance is defined as a fasting insulin level of ≥20 mU/L, with a peak insulin level of ≥150 mU/L during OGTT; severe insulin resistance is indicated by a fasting insulin level of ≥70 mU/L or a peak insulin level of ≥350 mU/L during OGTT)
6. Presence of extrapancreatic features

Out of these individuals, 138 underwent NGS and were enrolled in the study cohort, while genetic testing was declined by parents of the remaining 27 cases, as illustrated in Supplementary Fig. S1 and depicted in the Supplementary Data (18). The study protocol was approved by the Ethics Committees of Children's Hospital of Nanjing Medical University. Informed consent was obtained either from the patient or their legal guardians. Clinical and genetic features of a fraction of these patients were previously reported (5).

### Molecular Analysis and Variant Assessment

The identification of the causative variants was performed by means of NGS. Sixty-eight probands were screened by a NGS-based panel including 115 genes causative of dysglycemia (Supplementary Table S1) (18). All targeted regions including exons and exon–intron boundaries (includes 50 base pairs at each end) of the 115 genes were captured using a GenCap kit (MyGenostics GenCap Enrichment technologies). The enrichment libraries were sequenced on an Illumina HiSeq 2500 sequencer for paired reads of 150 bp. Seventy probands underwent WES testing. The WES procedure was performed as previously described (19). In brief, genomic DNA was extracted from blood lymphocytes and then underwent exome capture using the xGen Exome Research Panel v1.0 probe sequence capture array from IDT (Integrated Device Technology, USA), followed by NGS on the Illumina novaseq (Illumina, USA) platform. Variants with a quality score <20 (Q20) were filtered out, and the sequencing reads were aligned to the GRCh37/Hg19 reference genome using Burrows-Wheeler Aligner software. Filtering was conducted to retain only alleles with a minor allele frequency < 0.1%. Minor allele frequency was estimated by integrating all accessible data from the 1000 Genomes Project, dbSNP145, gnomAD, and the Exome Variant Server project. Filtered candidate variants were confirmed by Sanger sequencing. Exonic and intronic variants that were synonymous or not located within splice-site regions were excluded. The retained variants, comprising nonsynonymous variants and strong splice-site variants, were subsequently assessed using the American College of Medical Genetics and Genomics criteria and confirmed through direct Sanger sequencing. All the identified variants were checked for novelty utilizing free public HGMD, ClinVar, gnomAD, dbSNP, and Varsome. The absence of the variant in the HGMD, ClinVar, dbSNP, Varsome, and Gnomad databases and in available publications was used as a criterion for assessing the variant as a “novel mutation.” The potential pathogenic role of all the identified variants was predicted by SIFT, Mutation Taster, and PolyPhen-2. The in silico tool SpliceAI was used to predict the effect of a candidate variant on splicing.

### Web Resources

HGMD (http://www.hgmd.cf.ac.uk/ac/index.php)

ClinVar (https://www.ncbi.nlm.nih.gov/clinvar/)

gnomAD (http://gnomad.broadinstitute.org/)

dbSNP (https://www.ncbi.nlm.nih.gov/projects/SNP/)

Varsome (https://varsome.com/)

SIFT (https://sift.bii.a-star.edu.sg/)

Mutation Taster (http://www.mutationtaster.org/)

PolyPhen-2 (http://genetics.bwh.harvard.edu/pph2)

FSplice (http://www.softberry.com)

SpliceAI Predictor (https://spliceailookup.broadinstitute.org/)

### Statistical Analysis

For the statistical analysis, IBM SPSS version 21.0 (Armonk, NY, USA) was used. Normality was checked using the Shapiro–Wilk test. The Kruskal–Wallis test was used for the comparison of non-normal continuous data between 3 or more groups. Categorical data was analyzed using Fisher's exact test or chi-square test. *P* < .05 was considered statistically significant.

## Results

### General Characteristics of Cohort

**Figure 1.**
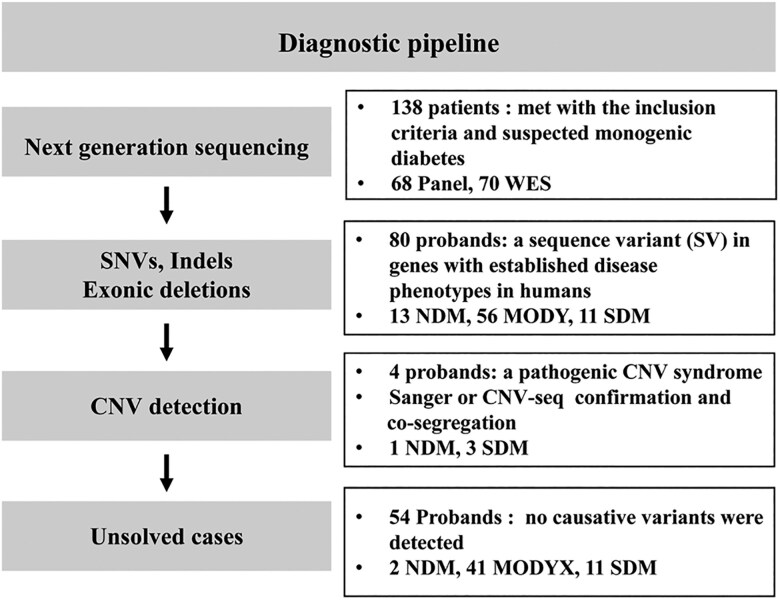
The overall workflow of the diagnostic pipeline in the study. Abbreviations: CNV, copy number variation; Indel, insertion/deletion; MODY, maturity-onset diabetes of the young; NDM, neonatal diabetes; Panel, dysglycemia gene panel sequencing; SDM, syndromic forms of diabetes mellitus; SNV, single nucleotide variant; WES, whole-exome sequencing.

**Figure 2.**
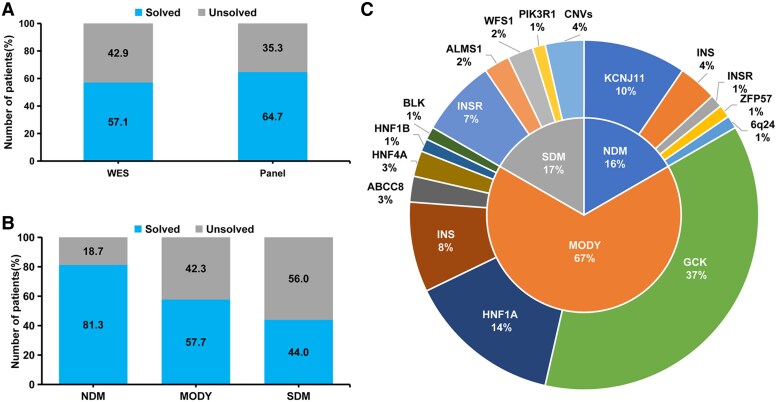
Diagnostic yield and monogenic variants inheritance in 138 patients with suspected monogenic diabetes. (A) Distribution of the genetic sequencing methods among patients with suspected monogenic diabetes. (B) Number of positive and negative cases among patients with different suspected monogenic diabetes types. (C) Genetic etiology of solved cases in our study. A total of 80 patients were identified to carry causative sequence variants distributed among 13 genes, with an additional finding of 4 probands harboring copy number variations.

A total of 138 pediatric patients with suspected MD were enrolled in the cohort (Fig. 1 and Table 1). Descriptions as well as a comparison of the different clinical parameters among and between patients with NDM, MODY, and SDM are provided in Table 1. Among the 138 patients, 16, 97, and 25 patients with NDM, suspected MODY, and SDM were assessed, respectively, either by targeted panel sequencing or WES (Fig. 2A). Causative variants were identified in 80 patients, with a pick-up rate of 58% (80/138) including positive test results of 81.3%, 57.7%, and 44% in patients with NDM, suspected MODY, and SDM, respectively (Fig. 2B). These variants were distributed among 13 genes (Fig. 2C). The 13 novel variants identified in this study are shown in Supplementary Table S2 (18). Additionally, 4 probands were found to harbor copy number variants (CNVs) through the incorporation of coverage analysis into WES (Supplementary Table S3) (18). Seventy-five patients (75/138, 54.3%) had a family history of diabetes. Accordingly, when exploring the distribution of family history among different forms of MD, *GCK*-MODY had the highest proportion of cases with family history (Table 2). Thirty-nine (28.3%) patients presented with diabetic ketoacidosis (DKA) or ketosis (DK) at onset. Distribution of DK/DKA was the most remarkable among NDM, followed by negative cases. *INS*-MODY was the only MODY type presented with DK/DKA. DK/DKA also was not observed in patients with *INSR* mutations. Within the cohort, 55 patients were treated with insulin therapy, 32 were prescribed metformin, 11 received sulfonylureas, and 44 were advised on diet and exercise. Notably, 19 patients shifted from insulin treatment to oral medications or lifestyle adjustments after receiving a specific genetic diagnosis for diabetes.

**Table 1.** Clinical characteristics of the 138 pediatric patients with suspected monogenic diabetes

	Overall n = 138	NDM n = 16	MODY n = 97	SDM n = 25	*P*-value
Sex: M/F	76/62	10/6	56/41	10/15	.2
Age at diagnosis (yr)	8.12 ± 4.72	0.84 ± 2.38	8.63 ± 4.27	10.81 ± 2.54	<.001
BW (kg)	3.22 ± 0.58	2.61 ± 0.64	3.31 ± 0.54	3.27 ± 0.49	<.001
Family history of DM, n (%)	75/138 (54)	1/16 (6.3)	69/97 (71)	5/25 (20)	<.001
Extrapancreatic features, n (%)	39/138 (28)	5/16 (31)	9/97 (9.3)	25/25 (100)	<.001
DK or DKA, n (%)	39/138 (28)	8/16 (50)	25/97 (26)	6/25 (24)	.14
HbA1c (%)	9.76 ± 3.39	8.52 ± 3.61	9.96 ± 3.36	9.63 ± 3.37	.4
Fasting glucose (mmol/L)	7.30 ± 3.24	11.07 ± 3.92	6.98 ± 2.75	6.62 ± 3.43	<.001
2-hour glucose*^a^* (mmol/L)	14.86 ± 5.89	18.63 ± 5.39	14.84 ± 5.96	13.76 ± 5.46	.2
Fasting insulin (mU/L)	18.31 ± 93.11	95.55 ± 300.04	5.81 ± 7.22	28.23 ± 52.18	.012
2-hour insulin*^a^* (mU/L)	49.60 ± 126.56	6.45 ± 4.82	22.13 ± 37.09	169.72 ± 250.60	<.001
Fasting CP (nmol/L)	0.66 ± 1.38	1.04 ± 3.54	0.55 ± 0.84	0.87 ± 0.68	<.001
2-hour CP*^a^* (nmol/L)	1.72 ± 1.62	0.38 ± 0.53	1.45 ± 1.00	3.27 ± 2.64	<.001
Positive gene detection, n (%)	84/138 (61)	14/16 (88)	56/97 (58)	14/25 (56)	.074
Treatment					
insulin, n (%)	55/138 (40)	7/16 (44)	39/97 (40)	9/25 (36)	.9
OHA, n (%)	43/138 (31)	8/16 (50)	24/97 (25)	11/25 (44)	.033
Sulfonylurea, n (%)	11/138 (8.0)	8/16 (50)	3/97 (3.1)	0/25 (0)	<.001
Metformin, n (%)	32/138 (23)	0/16 (0)	21/97 (22)	11/25 (44)	.003
Diet alone, n (%)	44/138 (32)	0/16 (0)	39/97 (40)	5/25 (20)	.002

Comparisons of numerical variables among groups were carried out by the Kruskal–Wallis test. Comparisons of categorical variables among groups were carried out by the chi-squared test or Fisher's exact test. *P* < .05 was considered statistically significant.

Abbreviations: BMI, body mass index; BW, birthweight; CP, C-peptide; DK, diabetic ketosis; DKA, diabetic ketoacidosis; DM, diabetes mellitus; F, female; HbA1c, glycated hemoglobin; M, male; MODY, maturity-onset diabetes of the young; NDM, neonatal diabetes; OHA, oral hypoglycemic agents; SDM, syndromic forms of diabetes mellitus.

^*a*^Levels of the oral glucose tolerance test and steamed bread meal test.

**Table 2.** Clinical characteristics of pediatric patients with maturity-onset diabetes of the young subtypes and maturity-onset diabetes of the young X

Characteristic	GCK-MODY n = 31	Non-GCK-MODY n = 25	MODYX n = 41	*P*-value			
				Among groups	GCK-MODY vs MODYX	GCK-MODY vs Non-GCK-MODY	Non-GCK-MODY vs MODYX
Sex: M/F	20/11	17/8	19/22	.15	.294	1	.294
Age at diagnosis (yr)	6.62 ± 2.70	9.71 ± 4.30	9.49 ± 4.76	<.001	.009	.009	.835
BMI (kg/m^2^)	16.38 ± 2.93	19.44 ± 3.33	19.29 ± 2.79	<.001	.002	.002	.857
Overweight, n (%)	4/28 (14)	7/23 (30)	11/41 (27)	.3	.518	.518	.986
BW (kg)	3.26 ± 0.52	3.37 ± 0.64	3.30 ± 0.50	.7	.742	.742	.742
Family history of DM, n (%)	28/31 (90)	19/25 (76)	21/41 (51)	.001	.003	.278	.123
DK or DKA, n (%)	0/31 (0)	3/25 (12)	22/41 (54)	<.001	<.001	.166	.003
HbA1c (%)	6.36 ± 0.37	10.80 ± 3.26	11.72 ± 2.70	<.001	<.001	<.001	.158
Fasting glucose (mmol/L)	6.00 ± 0.71	6.87 ± 3.68	7.67 ± 2.86	.043	.048	.271	.271
2-hour glucose*^a^* (mmol/L)	9.02 ± 1.62	15.25 ± 5.19	18.40 ± 5.22	<.001	<.001	<.001	.01
△2-hour glucose*^a^* (mmol/L)	3.02 ± 2.09	7.87 ± 6.46	10.73 ± 4.42	<.001	<.001	.001	.019
Fasting insulin (mU/L)	3.73 ± 3.99	6.88 ± 9.04	6.62 ± 7.69	.2	.214	.214	.893
2-hour insulin*^a^* (mU/L)	19.92 ± 25.18	25.78 ± 22.60	21.78 ± 47.88	.3	.846	.846	.846
Fasting CP (nmol/L)	0.35 ± 0.23	0.56 ± 0.43	0.67 ± 1.17	.11	.384	.594	.616
2-hour CP (nmol/L)*^a^*	1.47 ± 0.83	1.78 ± 1.03	1.28 ± 1.07	.056	.437	.437	.206
TG (mmol/L)	0.65 ± 0.26	1.53 ± 1.14	1.80 ± 1.82	<.001	.005	.054	.482
TC (mmol/L)	4.06 ± 0.74	4.38 ± 0.83	4.20 ± 1.22	.5	.578	.578	.578
HDL (mmol/L)	1.51 ± 0.36	1.24 ± 0.41	1.10 ± 0.34	<.001	<.001	.024	.16
Treatment at time of visit							
Insulin, n (%)	0/31 (0)	10/25 (40)	29/41 (71)	<.001	<.001	.001	.027
OHA, n (%)	0/31 (0)	12/25 (48)	12/41 (29)	<.001	.004	<.001	.204
Sulfonylurea, n (%)	0/31 (0)	3/25 (12)	0/41 (0)	.016	—	.166	.166
Metformin, n (%)	0/31 (0)	9/25 (36)	12/41 (29)	.002	.004	.003	.766

Comparisons of numerical variables among groups were carried out by the Kruskal–Wallis test. Comparisons of categorical variables among groups were carried out by the chi-squared test or Fisher's exact test. Post hoc pair-wise comparison was conducted using *t*-test for numerical variables and chi-squared test for categorical variables, with corrections for multiple testing using the false discovery rate. *P* < .05 was considered statistically significant.

Abbreviations: BMI, body mass index; BW, birthweight; CP, C-peptide; DK, diabetic ketosis; DKA, diabetic ketoacidosis; DM, diabetes mellitus; GCK-MODY, MODY subtype caused by GCK gene mutations; HbA1c, glycated hemoglobin; HDL, high-density lipoprotein; MODY, maturity-onset diabetes of the young; MODYX, suspected MODY patients without a known genetic cause of MODY; NDM, neonatal diabetes; non-GCK-MODY, MODY subtype caused by mutations in genes other than GCK; OHA, oral hypoglycemic agents; TC, total cholesterol; TG, triacylglycerol.

^*a*^Levels of the oral glucose tolerance test and steamed bread meal test.

### Clinical and Molecular Characteristics of Patients With MODY

Among the 97 patients with suspected MODY, the median age at diagnosis was 8.63 ± 4.27 years. Seven patients had extrapancreatic features, including short stature, anourethral atresia, and renal and cardiac anomalies. Twenty-five patients (25/97, 25.8%) were diagnosed with DKA or DK at the initial evaluation. Sixty-nine patients (69/97, 71.1%) had a family history of diabetes (Table 1). The MODY subtypes were identified in 56 patients (56/97, 57.7%): *GCK*-MODY in 31 (55.4%), *HNF1A*-MODY in 12 (21.4%), *INS*-MODY in 7 (12.5%), *HNF4A*-MODY in 2 (3.6%), *ABCC8*-MODY in 2 (3.6%), *HNF1B*-MODY in 1 (1.8%), and *BLK*-MODY in 1 (1.8%) patient (Fig. 2C). A total of 50 pathogenic variants were detected in 56 patients. Five variants (10%, 5/50) were novel and highly likely to be pathogenic (Table 3). We compared age at onset, sex distribution, birthweight, BMI, HbA1C, fasting blood glucose, fasting insulin, fasting C-peptide, 2 hours postload blood glucose, 2 hours postload blood insulin, 2 hours postload blood C-peptide, TG, TC, HDL, and the use of different treatment strategies according to the genetic etiology of patients. Since *GCK*-MODY has the highest proportion and shows distinct features from other MODY types, we divided all MODY patients into 3 groups: *GCK*-MODY, non-*GCK*-MODY, and MODYX (Table 2). Age at onset [H(2) = 15.97, *P* = <.001), BMI (H(2) = 14.6, *P* = <.001], percentage of patients with DK/DKA (*P* = <.001), HbA1C [H(2) = 48.57, *P* = <.001], fasting glucose [H(2) = 6.307, *P* = .043], 2-hour postload glucose [H(2) = 47.74, *P* = <.001], △2-hour glucose [H(2) = 44.38, *P* = <.001], TG [H(2) = 20.59, *P* = <.001], and percentage of patients receiving insulin (*P* = <.001) and oral hypoglycemic agents (*P* = <.001) were significantly lower in the *GCK*-MODY group when compared to both non-*GCK*-MODY and MODYX groups. In contrast, percentage of patients with family history of diabetes (*P* = <.001), HDL levels [H(2) = 16.61, *P* = <.001] and percentage of patients managed with diet alone (*P* = <.001) were significantly higher in the *GCK*-MODY group than the non-*GCK*-MODY and MODYX groups. In comparison to non-GCK-MODY patients, those with MODYX displayed increased 2-hour postload blood glucose levels (*P* = .01), △2-hour glucose (*P* = .019), a greater susceptibility to DK/DKA (*P* = .003), and more frequent usage of insulin injections (*P* = .027).

**Table 3.** Single nucleotide variants were identified in 80 cases with monogenic diabetes

Patient ID	Clinical phenotype	Gene	Accession number	Nucleotide change	AA change	Chromosomal location	Exon	Type of mutation	Mode of inheritance	Parental origin	Novelty	ACMG criteria
1-3	MODY	*GCK*	NM_000162.5	c.106C > T	p.R36W	chr7:44193002	2	Missense	AD	F, F, M	Known	LP
4	MODY	*GCK*	NM_000162.5	c.110T > C	p.M37T	chr7:44192998	2	Missense	AD	M	Known	LP
5	MODY	*GCK*	NM_000162.5	c.130G > A	p.G44S	chr7:44192978	2	Missense	AD	M	Known	P
6	MODY	*GCK*	NM_000162.5	c.238G > A	p.G80S	chr7:44191995	3	Missense	AD	F	Known	LP
7	MODY	*GCK*	NM_000162.5	c.544G > A	p.V182M	chr7:44189603	5	Missense	AD	M	Known	P
8	MODY	*GCK*	NM_000162.5	c.563C > T	p.A188V	chr7:44189584	5	Missense	AD	M	Known	LP
9	MODY	*GCK*	NM_000162.5	c.571C > T	p.R191W	chr7:44189576	5	Missense	AD	ND	Known	P
10	MODY	*GCK*	NM_000162.5	c.595G > T	p.V199L	chr7:44189443	6	Missense	AD	M	Known	LP
11	MODY	*GCK*	NM_000162.5	c.612T > A	p.N204K	chr7:44189426	6	Missense	AD	M	Known	LP
12	MODY	*GCK*	NM_000162.5	c.617C > T	p.T206M	chr7:44189421	6	Missense	AD	M	Known	P
13, 14	MODY	*GCK*	NM_000162.5	c.661G > A	p.E221K	chr7:44189377	6	Missense	AD	M, F	Known	P
15	MODY	*GCK*	NM_000162.5	c.667G > A	p.G223S	chr7:44189371	6	Missense	AD	M	Known	P
16	MODY	*GCK*	NM_000162.5	c.703A > T	p.M235L	chr7:44187409	7	Missense	AD	F	Known	LP
17	MODY	*GCK*	NM_000162.5	c.763A > G	p.T255A	chr7:44187349	7	Missense	AD	F	Known	LP
18	MODY	*GCK*	NM_000162.5	c.766G > A	p.E256K	chr7:44187346	7	Missense	AD	F	Known	P
19	MODY	*GCK*	NM_000162.5	c.892A > G	p.M298V	chr7:44186189	8	Missense	AD	M	Known	LP
20	MODY	*GCK*	NM_000162.5	c.1130G > T	p.R377L	chr7:44185219	9	Missense	AD	M	Known	P
21	MODY	*GCK*	NM_000162.5	c.1142T > C	p.M381T	chr7:44185207	9	Missense	AD	M	Known	P
22	MODY	*GCK*	NM_000162.5	c.1358C > T	p.S453L	chr7:44184775	10	Missense	AD	M	Known	P
23	MODY	*GCK*	NM_000162.5	c.1396T > C	p.X466R	chr7:44186165- 44186173	10	Missense	AD	F	Known	LP
24	MODY	*GCK*	NM_000162.5	c.449_451delTCT	p.F150del	chr7:44190587_44190589	4	Deletion	AD	F	Known	LP
25	MODY	*GCK*	NM_000162.5	c.641_654dup ACTACGAAGACCA	p.Q219Tfs*11	chr7:44189397-44189384	6	Duplication	AD	F	Novel	LP
26	MODY	*GCK*	NM_000162.5	c.908_916delGGCTTGTGC	p.R303-306delinL	chr7:44186173-44186165	8	Frameshift	AD	F	Novel	LP
27	MODY	*GCK*	NM_000162.5	c.1182_1195del	p.E395Gfs*59	chr7:44185154-44185167	9	Deletion	AD	De novo	Known	P
28	MODY	*GCK*	NM_000162.5	c.1278_1286del CGTGCGCAG	p.S426_R428del	chr7:44184855-44184847	10	Deletion	AD	M	Known	LP
29, 30	MODY	*GCK*	NM_000162.5	del 8-10	—	chr7:44184738-44186217	8-10	Deletion	AD	ND, ND	Known	P
31	MODY	*GCK*	NM_000162.5	c.1019 + 21(IVS8)G > A	Unknown	chr7:44186041	Intron 8	Splicing	AD	M	Known	VUS
32	MODY	*HNF1A*	NM_000545	c.44C > T	p.A15V	chr12:121416615	1	Missense	AD	F	Known	LP
33	MODY	*HNF1A*	NM_000545	c.335C > T	p.P112L	chr12:121426644	2	Missense	AD	F	Known	P
34	MODY	*HNF1A*	NM_000545	c.476G > A	p.R159Q	chr12:121426785	2	Missense	AD	M	Known	P
35	MODY	*HNF1A*	NM_000545	c.608G > A	p.R203H	chr12:121431404	3	Missense	AD	M	Known	P
36	MODY	*HNF1A*	NM_000545	c.775G > T	p.V259F	chr12:121432028	4	Missense	AD	M	Known	LP
37	MODY	*HNF1A*	NM_000545	c.815G > A	p.R272H	chr12:121432068	4	Missense	AD	F	Known	P
38	MODY	*HNF1A*	NM_000545	c.827C > A	p.A276D	chr12:121432080	4	Missense	AD	M	Known	P
39	MODY	*HNF1A*	NM_000545	c.1340C > T	p.P447L	chr12:121435307	7	Missense	AD	F	Known	P
40	MODY	*HNF1A*	NM_000545	c.872_873insC	p.G292fs*25	chr12:121432125-121432126	4	Frameshift	AD	De novo	Known	P
41	MODY	*HNF1A*	NM_000545	c.942delC	p.S315Vfs*27	chr12:121432194-121432195	4	Frameshift	AD	M	Novel	LP
42	MODY	*HNF1A*	NM_000545	c.1028_1029delCA	p.T343fs*75	chr12:121434137-121434138	5	Deletion	AD	M	Known	LP
43	MODY	*HNF1A*	NM_000545	c.1192C > T	p.Q398*	chr12:121434428	6	Nonsense	AD	M	Known	LP
44	MODY	*INS*	NM_000207.3	c.4G > A	p.A2T	chr11:2182198	2	Missense	AD	M	Known	LP
45	MODY	*INS*	NM_000207.3	c.94G > A	p.G32S	chr11:2182108	2	Missense	AD	De novo	Known	P
46	MODY	*INS*	NM_000207.3	c.269G > T	p.G90V	chr11:2181146	3	Missense	AD	F	Known	LP
47	MODY	*INS*	NM_000207.3	c.284G > A	p.C95Y	chr11:2181131	3	Missense	AD	De novo	Known	P
48, 49, 50	MODY	*INS*	NM_000207.3	c.188-31(IVS2)G > A	p.V63-Efs*78	chr11:2181258	Intron 2	Splicing	AD	De novo, M, de novo	Known	P
51	MODY	*HNF4A*	NM_000457	c.266G > A	p.R89Q	chr20:43034848	2	Missense	AD	M	Known	P
52	MODY	*HNF4A*	NM_000457	c.419G > A	p.R140Q	chr20:43042367	4	Missense	AD	M	Known	VUS
53	MODY	*ABCC8*	NM_001351295	loss1(:23-28) c.2329T > C	- p.W777R	chr11:17426058-17430064 chr11:17436120	23-28 19	Deletion Missense	AD,AR	M F or De novo	Novel Novel	LP VUS
54	MODY	*ABCC8*	NM_000352	c.4544C > T	p.T1515M	chr11:17415814	37	Missense	AD	M	Known	LP
55	MODY	*HNF1B*	NM_000458.4	c.457C > A	p.H153N	chr17:36099518	2	Missense	AD	M	Known	P
56	MODY	*BLK*	NM_001715.2	c.809C > T	p.T270M	chr8:11414203	9	Missense	AD	M	Known	VUS
57-59	NDM	*KCNJ11*	NM_000525.4	c.149G > A	p.R50Q	chr11:17409490	1	Missense	AD	ND, de novo, de novo	Known	P
60	NDM	*KCNJ11*	NM_000525.4	c.148C > G	p.R50G	chr11:17409491	1	Missense	AD	De novo	Known	P
61	NDM	*KCNJ11*	NM_000525.4	c.157G > C	p.G53R	chr11:17409482	1	Missense	AD	De novo	Known	LP
62	NDM	*KCNJ11*	NM_000525.4	c.176T > C	p.V59A	chr11:17409463	1	Missense	AD	De novo	Known	P
63	NDM	*KCNJ11*	NM_000525.4	c.511A > G	p.T171A	chr11:17409128	1	Missense	AD	De novo	Novel	LP
64	NDM	*KCNJ11*	NM_000525.4	c.685G > A	p.E229K	chr11:17408954	1	Missense	AD	De novo	Known	P
65	NDM	*INS*	NM_000207.3	c.128G > A	p.C43Y	chr11:2182074	2	Missense	AD	De novo	Known	P
66	NDM	*INS*	NM_000207.3	c.265C > T	p.R89C	chr11:2181150	3	Missense	AD	De novo	Known	P
67	NDM	*INS*	NM_000207.3	c.287G > A	p.C96Y	chr11:2181128	3	Missense	AD	De novo	Known	P
68	NDM SDM	*INSR*	NM_000208	c.3355C > T c.749-751delCCA	p.R1119W p.250-251del	chr19-7122904 chr19-7184549-7184552	18 3	Missense Deletion	AR AR	ND ND	Known Novel	LP VUS
69	NDM	*ZFP57*	NM_001109809	c.250 + 1delG c.805C > T	p.R269W	chr6:29643708-29643709 chr6:29641083	3 5	Splicing Missense	AR AR	M F	Known Known	LP VUS
70	SDM	*INSR*	NM_000208	c.2810C > T	p.T937M	chr19:7132201	14	Missense	AR	M and F	Known	LP
71	SDM	*INSR*	NM_000208	c.3472C > T	p.R1158W	chr19:7122682	19	Missense	AD	F	Known	LP
72	SDM	*INSR*	NM_000208	c.3697G > A	p.A1233T	chr19:7119557	21	Missense	AD	F	Novel	LP
73, 74	SDM	*INSR* *INSR*	NM_000208 NM_000208	c.3614C > T c.3670G > A	p.P1205L p.V1224M	chr19-7120676 chr19-7119584	20 21	Missense Missense	AR AR	M F	Known Known	LP LP
75	SDM	*INSR*	NM_000208	c.2314delG	p.V772fs*31	chr19:7143056	12	Frameshift	AD	F	Novel	LP
76	SDM	*WFS1* *WFS1*	NM_006005.3 NM_006005.3	c.2104G > A c.695_697dup	p.G702S p.R232dup	chr4:6303626 chr4:6293709-6293710	8 6	Missense Duplication	AR AR	F M	Known Novel	LP LP
77	SDM	*WFS1* *WFS1*	NM_006005 NM_006005	c.1525_1539delGTCTACC TGCTCTAT c.1372G > T	p.V509_Y513del p.A458S	chr4:6303046-6303061 chr4:6302894	8 8	Deletion Missense	AR AR	F M	Known Novel	LP VUS
78	SDM	*ALMS1* *ALMS1*	NM_015120.4 NM_015120.4	c.6322C > T c.6811G > T	p.Q2108* p.E2271*	chr2:73679973 chr2:73680462	8 8	Nonsense Nonsense	AR AR	F M	Known Novel	P P
79	SDM	*ALMS1* *ALMS1*	NM_015120 NM_015120	c.10616_10619delCAGA c.7664T > A	p.T3539fs*5 p.L2555*	chr2:73799622-73799626 chr2:73682415	16 9	Frameshift Nonsense	AR AR	F M	Known Novel	P P
80	SDM	*PIK3R1*	NM_181523	c.1945C > T	p.R649W	chr5:67592129	15	Missense	AR	De novo	Known	P

Abbreviations: AA, amino acid; ACGM, American College of Medical Genetics and Genomics; AD, autosomal-dominant; F, female; LP, likely pathogenic; M, male; MODY, maturity-onset diabetes of the young; ND, not determined; NDM, neonatal diabetes mellitus; P, pathogenic; SDM, syndromic forms of diabetes mellitus; VUS, variant of uncertain significance.

The clinical characteristics of *GCK*-MODY are shown in Table 2. Twenty-seven heterozygous mutations were identified in 31 unrelated probands: 12 pathogenic, 14 likely pathogenic, and 1 insignificant variant (Table 3). The c.1019 + 21G > A splice-site variant, which was described previously (20), was detected in a patient with a classical *GCK*-MODY phenotype and a family history spanning 3 generations. The c.1019 + 21G > A variant was not found in the gnomAD population database. According to the splice-site predictor SpliceAI, the intronic *GCK* c.1019 + 21 G > A variant is predicted to affect mRNA splicing, with a delta score (donor loss) of 0.71 and a delta score (acceptor gain) of 0.53. The most frequent variant was c.106C > T (p.R36W) (9.68%; 3/31 patients). Two variants (c.908-916delGGCTTGTGC and c.641-654dupACTACGAAGACCA) were novel. Novel c.641-654dupACTACGAAGACCA (p.Q219Tfs*11) variant causes termination of transcription in exon 3. A novel frameshift c.908-916delGGCTTGTGC (p.R303-L306delinsL) variant was found in the proband and in his father who also had mild diabetes. After genetic diagnosis, 4 patients with *GCK*-MODY ceased oral hypoglycemic agent, and 1 patient discontinued insulin treatment. All *GCK*-MODY patients achieved good glycemic control through adherence to dietary and exercise regimens.

Eight missense, 2 frameshift, 1 deletion, and 1 nonsense mutation were identified in 12 patients with *HNF1A*-MODY [median age: 10.34 years (9.96-12.25 years); male:female = 1:1], of which 7 were pathogenic and 5 were likely pathogenic variants. c.942delC(p.S315Vfs*27) variant was novel. A patient harboring the c.775G > T (p.V259F) variant displayed abnormal kidney development: ectopia of left kidney and right duplicated kidney. Patient with c.1192C > T (p.Q398*) variant had a history of surgery due to double outlet right ventricle. An 11-year-old female and a 7-year-old male, both having obesity, exhibited asymptomatic hyperglycemia and had a family history of diabetes spanning 3 generations. They were found to have pathogenic c.266G > A (p.R89Q) and variant of unknown significance c.419G > A (p.R140Q) variants in the *HNF4A* gene, respectively. Among 12 patients with *HNF1A*-MODY, 5 remained on metformin treatment, 3 transitioned from insulin to sulfonylurea treatment, 1 was lost to follow-up, and 3 adopted dietary and exercise therapy. In the case of *HNF4A*-MODY, 1 transitioned from insulin treatment to sulfonylurea medication and 1 was managed with dietary and exercise therapy.

Four missense and 1 splice-site variant of the *INS* gene were found in 7 patients [median age: 4.58 years (0.67-14.33 years); 3 patients were infants; all probands were male]. Pathogenic c.188-31(IVS2)G > A variant was detected in 3 unrelated probands. Six out of 7 (85.7%) of *INS*-MODY patients sought medical attention due to either presenting symptoms or DKA and were treated with insulin. In contrast, 1 patient with the p.A2T (c.4G > A) variant exhibited preserved pancreatic islet β-cell function and achieved good glycemic control with oral metformin. *ABCC8* variants were identified in 2 probands. One patient, diagnosed with diazoxide-unresponsive hyperinsulinemic hypoglycemia from birth, subsequently developed diabetes mellitus at the age of 10 years due to compound heterozygous variants, involving the deletion of exon 23_28 and c.2329T > C (p.W777R) variants. He presented with the coexistence of hypoglycemia and postprandial hyperglycemic episodes and was managed with the lifestyle interventions. In another case, a 12-year-old boy was found to carry the c.4544C > T (p.T1515M) variant, with no reported history of hyperinsulinemic hypoglycemia. Upon the diagnosis of *ABCC8*-MODY, he was transitioned to glibenclamide treatment for 1 year. However, insulin replacement reinitiated due to blood glucose perturbations. A heterozygous variant c.457C > A (p.H153N) in the *HNF1B* gene was found in a 10-year-old girl and in her mother. The proband had insulin-dependent diabetes with extrapancreatic features including bilateral renal cysts and abnormal liver function. Her mother had gestational diabetes. The previously reported c.809C > T (p.T270M) variant in the *BLK* gene was found in a 13-year-old overweight male patient with autoantibody-negative hyperglycemia and a strong family history of diabetes, which includes his mother, maternal grandfather, all 5 sisters of his grandfather, and 2 cousins of his mother. The patient with *BLK*-MODY was treated with a combination of metformin and insulin to achieve glycemic control.

### Clinical and Molecular Characteristics of Patients With Neonatal Diabetes

Sixteen patients (mean age: 0.22 ± 0.17 years; male:female = 10:6) were diagnosed with NDM, comprising 15 cases of permanent neonatal diabetes mellitus (PNDM) and 1 case of transient neonatal diabetes mellitus. NGS revealed that 14 (14/16, 87.5%) patients harbored causative variants. The distribution of the variants was *KCNJ11* in 8, *INS* in 3, *ZFP57* in 1, and *INSR* in 1 patient, respectively. In addition, we identified a patient with PNDM due to 750.44 kb-length paternal 6q24 duplication. Eight cases with NDM due to *KCNJ11* variants were treated with glibenclamide. Three cases caused by *INS* variants were treated with insulin. The patient who had paternal 6q24 duplication exhibited PNDM and was managed with a combination of glibenclamide and insulin. The infant diagnosed with transient neonatal diabetes mellitus due to *ZFP57* variants attained remission at 3 months of age without the need for any medication.

### Clinical and Molecular Characteristics of Patients With Syndromic Forms of Diabetes

Thirty-five patients were presented with extrapancreatic features in addition to hyperglycemia. Pathogenic variants were found in 24 patients, comprising 11 cases of SDM, 3 syndromic cases of NDM, 7 syndromic cases of MODY, and 3 cases with CNVs (Supplementary Table S3) (18). The extrapancreatic features of cases were shown in Supplementary Table S4 (18). One homozygous, 2 compound heterozygous, and 3e autosomal-dominant heterozygous variants were detected in the *INSR* gene of 6 patients, who were diagnosed with *INSR*-related severe insulin resistance syndrome. Two patients with c.3614C > T (p.P1205L) and c.3670G > A (p.V1224M) variants were siblings. Liver dysfunction and low HDL were presented in an overweight patient harboring c.2314delG (p.V772fs*31). A patient carrying c.1525_1539delGTCTACCTGCTCTAT (p.V509_Y513del) and c.1372G > T (p.A458S) variants at the *WFS1* gene had optic nerve atrophy. In patient 76, diabetes mellitus was the only presenting feature. Two patients with obesity fulfilled the diagnostic criteria for Alström syndrome. SHORT syndrome was diagnosed in patient 80 harboring heterozygous c.1945C > T (p.R649W) variant to the *PIK3R1* gene. Three patients were diagnosed with CNVs at 7q22, 16p11.2, and 17q12 with the help of NGS.

## Discussion

The prevalence and characteristics of MD among pediatric population of European ancestry are well described (1-3). However, only a few related studies are available from China (5, 21-31), as listed in Supplementary Table S5 and depicted in the Supplementary Data (18). In the current report, 138 Chinese children presenting with suspected MD were screened for the relevant genes. Our diagnostic yield of 58% is in agreement with the previous studies in the pediatric population of China (24, 26, 28). In our cohort, the distribution of MODY, NDM, and SDM among the positive cases were 66.67%, 16.67%, and 16.67%, respectively. Similar to multiple foreign and domestic studies (1, 24, 26, 28), *GCK* was the leading cause of MODY in the current study (31/56, 55.4%). *KCNJ11* was the commonest form of NDM in our nonconsanguineous population group, which is in line with the recent international cohort study (32). Unlike the same study (32), no *ABCC8* variant was found. To date, at least 67 diabetes-related genetic syndromes have been described (33), of which extreme rarity makes the estimation of their prevalence in different populations difficult. In our single-center study, *INSR* was the major etiologic gene for SDM. Our study highlights that the prevalence of MD is more influenced by the preselection criteria and genetic screening methods rather than race, ethnicity, or geographical region. Fifty-eight cases (42%) of patients were identified without known pathogenic variants, suggesting potential novel forms of monogenic or oligogenic diabetes. The limitations of current technology may have contributed to this finding, highlighting the importance of comprehensive genetic methods such as WGS, RNA sequencing, and mitochondrial testing. Additionally, future research integrating metabolomics and transcriptomics analyses may elucidate novel mechanisms associated with atypical diabetes.

Family history of diabetes is the one of the most prominent features among our MODY patients, especially among patients harboring *GCK* variants (Table 2). *GCK*-MODY also differed significantly from the other subtypes of MODY with lower HbA1_C_, fasting C-peptide and 2-hour glucose levels and a diminished need for insulin according to our findings (Table 2) and previous reports (8, 20). However, the comparison of the clinical parameters was almost unremarkable between patients with MODY and MODYX (Table 2). Additionally, we did not detect any clinical or biochemical heterogeneity in relation to the mutation type or location in patients with *GCK*-MODY, while previous reports have suggested a correlation between genotype and phenotype in *HNF1A*-MODY (11) and *INS*-MODY (34). However, the number of non-*GCK*-MODY cases in our cohort were not sufficient to draw this conclusion confidently.

In our study, varying levels of lipid profiles were identified among patients with suspected MODY. Patients with *GCK*-MODY demonstrated decreased levels of TC and increased levels of HDL. Conversely, patients with non-*GCK*-MODY exhibited elevated levels of TC, whereas patients with MODYX displayed higher levels of TG. *HNF* family transcription factors and *GCK* have been reported to have a regulatory effect on lipid metabolism, in addition to playing a major role in glucose homeostasis (35-38). Biochemical assessment of *HNF4A*-null mice demonstrated that reduced *HNF4A* activity cause dyslipidemia through decreased circulating lipid levels and altered HDL composition, which resulted from the disruption of TG and TC secretion from the liver (35), albeit the lipid profile of the reported families with *HNF4A*-MODY varies considerably (39, 40). Cholesterol metabolism and HDL composition were also proved to be deranged by the lack of *HNF1A* and *HNF1B* activity in the gene knockout animals (36, 37). In fact, dyslipidemia frequently accompanies the mutations and the complete deletion of *HNF1B* (41). On the other hand, cardioprotective lipid profile was observed in *GCK*-MODY patients (38, 42). Supportively, glucokinase activators are known to raise serum TG levels (43). Low TG and low HDL levels have been reported in *GCK*-MODY patients, serving as markers to differentiate *GCK*-MODY from *HNF1A*-MODY (44, 45). In the current study, however, *GCK*-MODY patients displayed low TG levels and high HDL levels (Table 2). This discrepancy did not reach statistical significance when comparing the lipid profile of *GCK*-MODY exclusively with *HNF1A*-MODY patients (data not shown), and this was probably due to the small number of *HNF1A*-MODY subjects in our cohort. In general, long-term outcomes of lipid perturbations on metabolic diseases as well as on related morbidity and mortality in patients with *HNF4A-*MODY*, GCK-*MODY, *HNF1A-*MODY, and *HNF1B-*MODY require future investigations.

*INS*-MODY was the third most frequent cause of MODY in our cohort, despite it being generally considered a rare type of MODY (1). *INS*-MODY showed the most severe phenotype and in accordance with the previous studies (34). Our study is the first to report a pediatric patient (patient 44) with *INS*-MODY carrying the p.A2T (c.4G > A) mutation. Similar to documented adult cases of *INS*-MODY with p.A2T (c.4G > A) mutation, this patient can maintain stable blood glucose levels with oral medications, although a minority may require transition to insulin replacement therapy (46). Among individuals with *INS*-MODY, there was diversity in both the age of onset and clinical severity, even sharing identical mutations. Inactivating mutations in the *ABCC8* gene have been reported to be associated with hyperinsulinemic hypoglycemia in infancy and diabetes later in life, particularly in individuals with biallelic variants in the *ABCC8* gene (47, 48). Herein, we present a child (patient 53) who was diagnosed with hyperinsulinemic hypoglycemia at birth and then developed diabetes mellitus at the age of 10 years due to compound heterozygous variants (exon 23_28del and c.2329T > C (p.W777R)) of *ABCC8*. Lastly, *BLK* is linked to MODY in individuals with higher BMI (49), which is in consistent with the phenotype of our patient with a history of strong autosomal-dominant inheritance. *BLK* was suggested to be excluded from the MODY list owing to its lack of enrichment in the European MODY cohort and higher frequency in the nondiabetic European population (50). Association between *BLK* and MODY must be ascertained using large sample size in non-European ethnicities.

NDM arising from *6q24* abnormalities usually resolves by 18 months (29), To the best of our knowledge, there has been only 1 reported case of *6q24*-related PNDM (51). Intrauterine growth restriction and prematurity were also assigned to 6q24 abnormalities (52), which is also the case for our PNDM patient harboring paternal *6q24* duplication. Thus, PNDM should not rule out the etiology of *6q24* abnormalities, especially in the presence of intrauterine growth restriction history.

In a systematic review, 67 distinct syndromes associated with diabetes were identified, with diabetes being the primary clinical feature in 30 genetic syndromes. These syndromes are mainly characterized by the involvement of multiple systems, including the endocrine, nervous, visual, and auditory systems (33). In this study, the extrapancreatic features were primarily characterized by insulin resistance, short stature, intellectual disability, cardiac anomalies, and hearing abnormalities, with genetic pickup rates of 76.9%, 77.8%, 66.7%, 60%, and 60%, respectively. Homozygous or compound heterozygous mutations to the *INSR* gene leads to Donohue syndrome or to its milder form—Rabson–Mendenhall syndrome. On the other hand, type A insulin resistance caused by heterozygous variants, as the most benign manifestation, is characterized by insulin resistance, acanthosis nigricans, and hyperandrogenism that is usually identified peri- or postpubertally (53). Consistent with previous reports (54), the case (patient 75) with type A insulin resistance manifested obesity, potentially leading to misclassification as T2DM. The constellation of diabetes mellitus, diabetes insipidus, optic atrophy, and deafness gives rise to the phenotype of Wolfram syndrome (55). However, hyperglycemia precedes all other symptoms in some cases and makes the establishment of Wolfram syndrome diagnosis challenging (55). In this case, genetic diagnosis is the only tool that helps to reveal correct the etiology and warrants routine follow-ups for the anticipated manifestations.

In our cohort, after receiving a precise genetic diagnosis of MD, 19 patients (13.8%, 19/138) transitioned from insulin therapy to oral agents or lifestyle interventions. NGS helped switch the management of hyperglycemia from unnecessary insulin injections to glibenclamide in 8 of our patients with *KCNJ11* mutations. In 29 families of *GCK*-MODY, parents harboring the same genetic variants as the proband manifested with hyperglycemia and had been previously diagnosed with gestational diabetes or T2DM. As a result, genetic testing within the family members prevented unnecessary treatments for individuals with *GCK*-MODY. Among 11 patients with *HNF1A*-MODY, 3 patients transitioned from insulin to sulfonylurea, 5 were treated with metformin, and 3 utilized lifestyle interventions, although the ISPAD/IDF guidelines recommend first-line use of oral sulfonylurea for individuals with *HNF1A*-MODY. Our research indicated that most children with *HNF1A*-MODY still had good metabolic control with dietary intervention alone or oral administration of metformin, consistent with previous research findings (56). The clinical characteristics of most *INS*-MODY patients (75%, 6/8) exhibit similarities to T1DM. These patients persist with insulin therapy without altering their treatment regimen postgenetic diagnosis. Nevertheless, a precise genetic diagnosis can facilitate genetic counseling and evaluation of the recurrence risk for patients. Therefore, a positive result of genetic testing would replace the previous diagnosis of T1DM or T2DM with a diagnosis of MD, which in turn offers benefits for personalized treatment, evaluation of recurrence risk, and informed decision-making for future management.

## Summary

Due to overlaps in clinical and biochemical characteristics of patients with different types of MD, NGS, particularly WES, is considered the gold standard in the diagnosis of MD. Our study emphasized that the prevalence of MD is more influenced by the preselection criteria and genetic screening methods rather than race, ethnicity, or geographical region. Additionally, we highlighted the broad heterogeneity of the same pathogenic variants, which range from isolated hypoglycemia to isolated diabetes in different severity. The precise diagnosis of diabetes has enabled the replacement of unnecessary insulin injections with oral medications or lifestyle adjustments in our patients and their adult family members. In summary, this study described clinical, biochemical, and genetic characteristics of Eastern Chinese children suffering from MD and underscored the importance of NGS methods for better individualized management.

## Data Availability

Original data generated and analyzed during this study are included in this published article or in the data repositories listed in References.
